# The Berlin Tuberculosis Congress1Written at the request of Dr. Pannwitz, General Secretary of the Congress, and forwarded simultaneously to several American journals. The medical, veterinarian, and scientific press is requested to call the attention of its readers to this Congress.

**Published:** 1899-04

**Authors:** Ch. Wardell Stiles

**Affiliations:** Scientific Attaché, U. S. Embassy, Berlin, Germany


### COMMUNICATION.

### THE BERLIN TUBERCULOSIS CONGRESS.[1]

THE "German Central Committee for the Erection of Sanitaria for Consumptives" has issued a call for a Congress to be held in Berlin, Germany, May 24–27, 1899, for the purpose of discussing the subject of tuberculosis. This Congress will meet in the new building of the Imperial Diet, and is under the patronage of Her Majesty the Kaiserin, while Prince Hohenlohe, the Imperial Chancellor, will serve as Honorary President. All of the German States, also local authorities, medical faculties and societies, and all corporations interested in fighting tuberculosis, have been requested to send delegates. All foreign countries represented at the Imperial Court have been invited to take part. The United States Embassy has been requested to extend a cordial invitation to American physicians to become members of the Congress, and the same invitation has been extended through other missions to physicians of other nationalities.

As a basis for discussion papers will be presented as follows: "Distribution and Extent of Tuberculosis," by Geheimrath Koehler, Director of the Imperial Health Office, and Geheimrath Krieger, of Strasburg; "Etiology," by Professors Robert Koch and B. Fränkel, of Berlin; "Prophylaxis," by Professor Gerhardt and Generaloberartz Schjerning, of Berlin; "Therapy," by Professor von Ziemssen, of Munich, and Professor Schroetter, of Vienna; "Sanitaria," by Herr Gaebel, President of the Imperial Insurance Office, Berlin, and Dr. Dettweiler, of Falkenstein.

Following the presentation of the two leading papers (limited to twenty minutes each) in the respective divisions, there will be a general discussion, speakers being limited to ten minutes each. All papers and remarks are to be in German, although the chairman is empowered to make exceptions during the general discussion.

All persons interested in the subject of tuberculosis are eligible for membership. Membership cards (twenty marks, nearly five dollars) are to be obtained at the office of the Congress ("Bureau des Organisations-Komites, Wilhelm Platz 2, Berlin, W."), and entitle the holder to a copy of the *Proceedings*. An early registration is requested.

[1] Written at the request of Dr. Pannwitz, General Secretary of the Congress, and forwarded simultaneously to several American journals. The medical, veterinarian, and scientific press is requested to call the attention of its readers to this Congress.

The writer has been requested to furnish a list of Americans to whom special invitations to the Congress should be sent. He has complied with this request, so far as his personal and professional acquaintance with specialists in this line has permitted, and has also suggested to the committee that invitations be sent to the various medical societies and faculties. There are undoubtedly many American practitioners especially interested in tuberculosis and possibly some laboratory workers whom he has overlooked. Should any such person desire to attend the Congress, yet prefer to receive a personal invitation, the writer will be pleased to forward the name of such person, upon proper introduction, to the Executive Committee of the Congress. As "proper introduction" will be considered a letter from any recognized medical, scientific, or veterinary faculty or society.

CH. WARDELL STILES, PH.D.,
Scientific Attaché, U. S. Embassy, Berlin, Germany.

---

### SWINE NOTES.

It will pay to disinfect the hog-pens regularly during the summer by sprinkling carbolic acid liberally over the floor and sides.

No matter how small the farm, pigs may be profitably kept on food suitable to them that otherwise would probably be wasted.

A prominent breeder of hogs says that for ten years he has been able to dispose of his hogs at five dollars a hundred, and he has kept a careful record. Those who have stuck to the business right through have had but little reason to complain of the result. The average is good.

Give the hogs as good care as any other stock. Do not go on the theory that anything is good enough for a hog. Anything which comes handy for a feed will never bring the hogs to a profitable market. Dish-water is not a very hearty feed, neither is moldy corn as good as that which is sound.

It pays best to raise for market hogs of the best grades, and it pays to give them the treatment necessary for their best development all the time. This should be begun with the pigs through the sow from before the time she brings her litter, and continued without interruption. It is an actual loss to permit them at any time to become stunted. It is more than a loss of time. It takes more feed to start them again and much more after getting them started, for every pound added to their weight.

